# Liquid profiling of circulating tumor DNA in colorectal cancer: steps needed to achieve its full clinical value as standard care

**DOI:** 10.1002/1878-0261.13156

**Published:** 2021-12-20

**Authors:** Maren Hedtke, Rodrigo Pessoa Rejas, Matthias F. Froelich, Volker Ast, Angelika Duda, Laura Mirbach, Victor Costina, Uwe M. Martens, Ralf‐Dieter Hofheinz, Michael Neumaier, Verena Haselmann

**Affiliations:** ^1^ Institute of Clinical Chemistry Medical Faculty Mannheim of the University of Heidelberg University Hospital Mannheim Germany; ^2^ Department of Radiology and Nuclear Medicine University Medicine Mannheim Medical Faculty Mannheim of the University of Heidelberg Germany; ^3^ Cancer Center Heilbronn‐Franken SLK‐Clinics MOLIT Institute for Personalized Medicine Heilbronn Germany; ^4^ Day Treatment Center (TTZ) Interdisciplinary Tumor Center Mannheim (ITM) III Medical Clinic Medical Faculty Mannheim of the University of Heidelberg University Hospital Mannheim Germany

**Keywords:** colorectal cancer, ctDNA, liquid biopsy, liquid profiling, real‐world

## Abstract

The analysis of circulating tumor DNA (ctDNA) is at the threshold of implementation into standard care for colorectal cancer (CRC) patients. However, data about the clinical utility of liquid profiling (LP), its acceptance by clinicians, and its integration into clinical workflows in real‐world settings remain limited. Here, LP tests requested as part of routine care since 2016 were retrospectively evaluated. Results show restrained request behavior that improved moderately over time, as well as reliable diagnostic performance comparable to translational studies, with an overall agreement of 91.7%. Extremely low ctDNA levels at < 0.1% in over 20% of cases, a high frequency of concomitant driver mutations (in up to 14% of cases), and ctDNA levels reflecting the clinical course of disease were revealed. However, certain limitations hampering successful translation of ctDNA into clinical practice were uncovered, including the lack of clinically relevant ctDNA thresholds, appropriate time points of LP requests, and integrative evaluation of ctDNA, imaging, and clinical findings. In conclusion, these results highlight the potential clinical value of LP for CRC patient management and demonstrate issues that need to be addressed for successful long‐term implementation in clinical workflows.

## Graphical abstract

Liquid profiling (LP) is a promising approach for real‐time assessment of the cumulative tumor mutational profile from a single blood draw. Currently, the workflow is about to be implemented into standard of care for the management of patients with colorectal cancer (CRC), with the aim to better guide CRC diagnostics and optimize therapeutic decisions. Here we report our 5‐year, single medical center clinical experience in LP for CRC patient management.

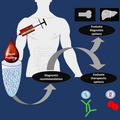

**Abbreviations**
BEAMing: beads, emulsification, amplification, and magnetics
*BRAF*
: B‐Raf proto‐oncogene, serine/threonine kinase
CAP: college of American Pathologists
cfDNA: cell‐free DNA
CI: confidence interval
COVID‐19: corona virus disease 2019
CR: complete response
CRC: colorectal cancer
CT: computed tomography
ctDNA: circulating tumor DNA
ddPCR: digital droplet PCR
EGFR: epithelial growth factor receptor
IQR: interquartile ratio
*KRAS*
: KRAS proto‐oncogene, GTPase
LP: liquid profiling
MAF: mutant allele frequency
mCRC: metastatic colorectal cancer
MRD: minimal residual disease
MRI: magnetic resonance imaging
MSI: microsatellite instability
NGS: next‐generation sequencing
*NRAS*
: NRAS proto‐oncogene, GTPase
PD: progressive disease
PR: partial response
qPCR: quantitative PCR
SD: stable disease
UMM: University Medical Center Mannheim

## 1 Introduction

Standard of care for colorectal cancer (CRC) patients to guide therapy selection involves tissue‐based genetic testing for at least three molecular biomarkers—*RAS* as a negative predictive marker for response to anti‐epithelial growth factor receptor (EGFR) antibodies; B‐Raf proto‐oncogene, serine/threonine kinase (*BRAF*) as a negative prognostic marker and to predict response to the combination treatment with BRAF inhibitors and anti‐EGFR monoclonal antibodies; and microsatellite instability (MSI) status to evaluate the efficacy of immune‐checkpoint inhibitors [1]. The genetic tumor landscape's importance for prognostic and therapeutic patient stratification is also reflected in the new consensus molecular subtypes of CRC [2]. However, tissue‐based testing is limited in that primary tumor tissue may not reflect current mutational status, while biopsies carry a general risk of complications, and may be unobtainable, particularly during follow‐up [3, 4].

Over the last few years, blood‐based liquid profiling (LP), commonly referred to as liquid biopsy, has emerged as a promising, minimally invasive tool for the diagnostic management of cancer patients. It is based on the detection of circulating tumor DNA (ctDNA) against a background of wild‐type DNA by identification of tumor‐derived genetic or epigenetic alterations. A single blood‐draw enables assessing the cumulative tumor mutational landscape in real‐time, thus doing justice to both intra‐ and intertumor heterogeneity [5, 6, 7]. LP can be used as a personalized molecular tumor marker in minimal residual disease (MRD) detection and for surveillance of cancer patients [8, 9, 10, 11], to monitor the tumor evolution under therapy, and to guide therapeutic decisions by detecting emerging targetable tumor alterations [12, 13, 14, 15]. Various translational studies have demonstrated LP's benefits for the management of CRC patients [16, 17, 18, 19, 20], for example, through lead‐time reduction for detection of disease recurrence compared to imaging of 2–15 months [21].

Taking into consideration these potential benefits for CRC patients, LP has recently been introduced into clinical management of CRC patients within clinical trials. However, successful implementation of a new biomarker into clinical care includes, among other issues, reimbursement, and incorporation into clinical practice guidelines [22], and is often slower than expected [23]. In case of LP, there is still a lack of standardized pre‐analytical and analytical procedures, as demonstrated by external quality assessment schemes (EQAs) [24]. Most of the countries' CRC guidelines have not yet included the tests for the applications described above, and most importantly, reimbursement options remain limited in several countries [25]. As a result, LP is not integrated into clinical care as one might expect.

In Germany, the University Hospital Mannheim was the first to establish LP for the detection of somatic mutations for CRC patients in 2016—at that time, the S3 guideline included LP‐based detection of *RAS* mutations as an alternative to tissue‐based testing if a biopsy is was infeasible [26]. The Institute of Clinical Chemistry was the first to obtain an ISO‐15189 accreditation for various LP assays, was nominated by the Reference Institute for Bioanalytics as a reference institute for ctDNA analysis, and as such is responsible for proficiency testing within Europe. Since limited data are available regarding diagnostic performance, clinician request behavior, and integration of LP for clinical decision making in routine clinical care of CRC patients, LP tests ordered between 2016 and 2021 in routine clinical practice at University Hospital Mannheim were evaluated and the results summarized in this manuscript to provide insights into the current status of implementation of LP in routine CRC patient care.

## 2 Material and methods

### 2.1 Scope and patients

Within this retrospective evaluation, all LP tests for CRC patients requested between September 2016 and January 2021 as part of routine clinical practice at the Institute of Clinical Chemistry, University Medical Centre Mannheim (UMM), University of Heidelberg, Germany, were retrospectively evaluated. Molecular pathology analysis of tumor tissue for method comparison with LP was performed by Sanger sequencing or next‐generation sequencing as part of standard care either at the UMM or at an external pathology department. The LP tests were requested for both inpatients and outpatients at UMM as well as by external hospitals. All patients suffered from histological confirmed CRC, and LP was performed as part of routine clinical testing, including written informed consent from patients. Additionally, conventional protein tumor markers CA 19‐9 and CEA were determined at the Institute for Clinical Chemistry for UMM patients, and radio‐imaging diagnostics were performed as clinically indicated at different diagnostic sites. Interval and frequency of diagnostic procedures and all clinical decisions based on diagnostic findings were at the discretion of the treating physicians and were made in accordance with current institutional and national guidelines. This retrospective evaluation was approved by the Institutional Review Board (2020‐868‐AF11) and conducted in accordance with the Declaration of Helsinki.

In total, 243 LP tests for 168 CRC patients were performed as part of standard care for *RAS* using beads, emulsification, amplification, and magnetics (BEAMing), for *BRAF* V600 by digital droplet PCR (ddPCR), or for both molecular targets. The analytical performance of all tests used for LP was evaluated and proven by regular, successful participation in EQA schemes as well as by obtaining a flexible accreditation for these assays according to DIN EN ISO‐15189.

### 2.2 Sample collection and processing

For each LP, 10–20 mL of blood was collected in cell‐free DNA (cfDNA) BCT CE tubes (Streck, Omaha, NE, USA), dispatched to the laboratory at ambient temperature, and processed within 72 h of blood collection. Upon sample receipt, plasma was separated by two consecutive centrifugation steps. First, blood samples were centrifuged at 1600 ***g*** for 10 min at room temperature without brakes. The supernatant was transferred to a new tube and centrifuged at 6000 ***g*** for 10 min at room temperature without brakes. Plasma was either used immediately for cfDNA isolation or stored at −80 °C.

Cell‐free DNA was isolated from 3 mL of plasma using the QIAamp Circulating Nucleic Acid Kit (Qiagen, Hilden, Germany) according to the manufacturer's instructions, except for an extended incubation time with proteinase K of 1 h instead of 30 min. For BEAMing, cfDNA was eluted in 140 µL of AVE buffer, and for ddPCR in 70 µL. The cfDNA was either used immediately for LP assays or stored at −20 °C for up to 7 days.

### 2.3 BEAMing

*RAS* mutational status for common somatic variations (codons 12, 13, 59, 61, 117, and 146 for *KRAS* and *NRAS*, respectively) was determined in cfDNA using the OncoBEAM® RAS CRC Kit (Sysmex Inostics, Hamburg, Germany) according to the manufacturer's instructions. Briefly, 140 µL of cfDNA and control samples (nontemplate control, positive control carrying specific mutations) were used in six multiples of 65‐µL reactions for target‐specific multiplex amplification PCR. Amplified PCR products were pooled, then diluted with a 1× pH 8.0 low‐EDTA TE buffer to obtain the optimal concentration for subsequent emulsion PCR. After emulsion PCR, the emulsion was broken to recover the amplicons bound to magnetic beads. This was followed by denaturation and hybridization of fluorescent‐labeled DNA probes (universal, wild type‐specific, mutant‐specific) to the single‐stranded amplicons bound to magnetic beads. The read‐out was performed by flow cytometry analysis on the CyFlow Cube 6i (Sysmex Inostics), and results were evaluated using the fcs express software (DeNovoSoftware, Pasadena, CA, USA). Identified mutations are indicated by the software together with the respective mutant allele frequency (MAF), absolute quantification is not included. The software detects insufficient DNA input (e.g., due to low cfDNA sample concentration) by the number of extended beads below a predefined cutoff, and these samples are marked as invalid. In such cases, blood sample collection and testing were repeated.

### 2.4 ddPCR

For the detection of *BRAF* V600 (including *BRAF* V600E, V600K, and V600R) in cfDNA, a ddPCR was performed using the ddPCR™ BRAF V600 Screening Kit (Bio‐Rad, Pleasanton, CA, USA) according to the manufacturer's instructions and as described previously [27]. In brief, 18–36 µL of isolated cfDNA was used in three to six multiples of 20‐µL reactions for emulsion PCR. The optimal annealing temperature of emulsion PCR was determined to be 54.5 °C. Droplets were generated by mixing 20 µL of ddPCR master mix with 70 µL of generation oil in a cartridge of an Automated Droplet Generator (QX200™; Bio‐Rad) and analyzed using the QX200™ Droplet Reader (Bio‐Rad). Results were evaluated using quantasoft analysis software version 1.7.4 (Bio‐Rad). Each run included one nontemplate control, one negative control (wild‐type), and one positive control (mutant). Validation studies revealed a limit of blank of < 1 copy·µL^−1^, a MAF of 0.05% as the limit of detection, and a MAF of 0.1% as the limit of quantification, based on a coefficient of variation < 25% for quantitative results, as recommended by the Guidelines for Validation of quantitative PCR‐based methods [28].

### 2.5 Imaging

Computed tomography (CT) examinations were performed with a multi‐detector CT scanner (Somatom Emotion or Somatom Flash, Siemens Healthineers, Erlangen, Germany) as part of standard CT protocols for CRC patients. CT scans were analyzed by the respective on‐call radiologist and reviewed by a consultant radiologist. Imaging studies were reviewed according to Response Evaluation Criteria in Solid Tumors (recist), version 1.1 [29], and a clinical significant response was defined as a complete response (CR), partial response (PR), or stable disease (SD).

### 2.6 Statistical analysis

The results of the data analysis are presented as descriptive statistics including mean, median, 95% confidence intervals (95% CI), and the interquartile ratio (IQR) where applicable. The positive, negative, and overall percentage agreement were calculated for LP testing using the tumor mutational status in tissue as a reference. Additionally, intertest agreement was assessed using Cohen's kappa coefficient, while between group differences were assessed by Student's *t*‐test or Fisher's exact test, two‐tailed. For all statistical analyses, *P*‐values < 0.05 were considered as statistically significant.

All statistical analyses and graph plotting were carried out using graphpad Software (GraphPad, San Diego, CA, USA) and r version 4.1.0 (https://www.r‐project.org) or Excel (version 2019, Microsoft Corporation, Redmond, WA, USA).

## 3 Results

### 3.1 Demographics and patient characteristics

**Fig. 1:**
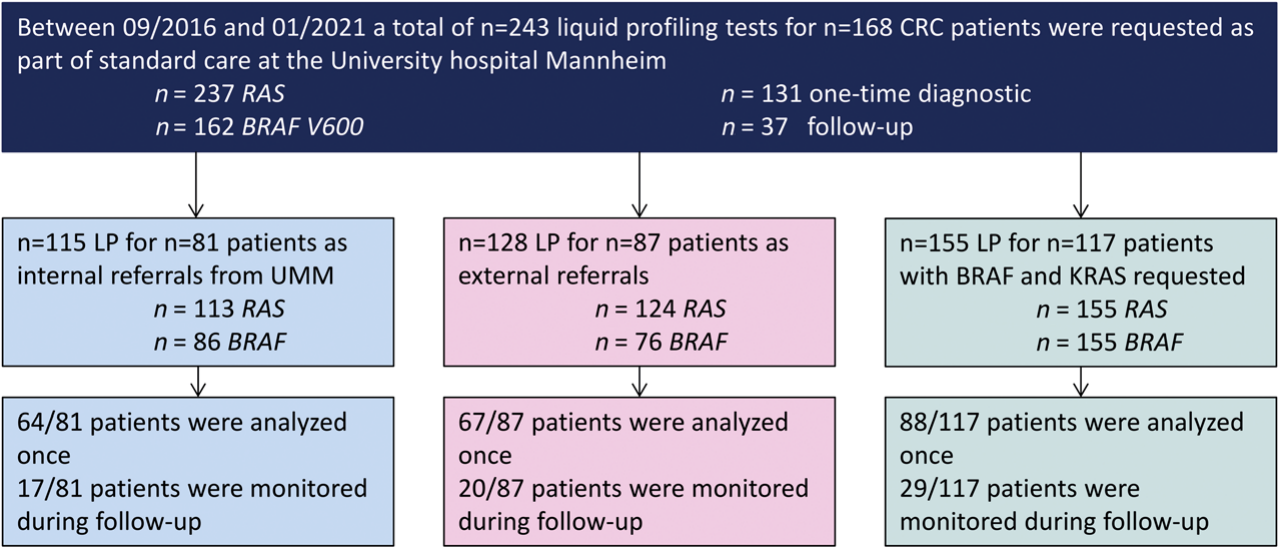
Evaluation of LP tests requested for CRC patients. The flow diagram displays the number of patients and LP tests ordered for *RAS* and/or *BRAF* V600 between September 2016 and January 2021 in our laboratory as part of standard care and the strategy used for data evaluation. Specifically, 243 LP tests were performed for a total of 168 patients diagnosed with CRC. The number of patients and tests ordered as internal requests from various departments within our hospital vs external referrals from other hospitals is presented, as is the respective information for all patients for whom both analytes were determined.

A total of 243 LPs for *RAS* and/or *BRAF* V600 requested at UMM for 168 CRC patients between September 2016 and February 2021 were retrospectively evaluated. Figure 1 provides an overview of evaluation and data analysis strategy. 115/243 (47.3%) LPs were performed for inpatients or outpatients at UMM, and 128/243 (52.6%) were requested by external hospitals nationwide. Overall, clinical information was not available for 38/168 (22.6%) patients. In cases where clinical information was obtainable either from the hospital information system or from a doctor's letter, the majority of patients suffered from stage IV CRC (54.8%), with ages ranging from 33 to 92 years, and 59/168 (35.1%) being female. 131/168 (77.9%) patients received one LP test, whereas 37 patients (22%) were monitored during their disease course. Information on patients' current treatment was available for 178/243 (73.3%) requested LPs. In detail, 44 patients were either treatment‐naïve or received no therapy, 48 received chemotherapy, 80 received a targeted therapy, and five were treated with an immune‐checkpoint inhibitor. Patient characteristics and information on the administered therapies are summarized in Table 1, while more detailed information is provided in Table S1.

**Table 1:** Patient characteristics and LP requesting behavior.

	Total	Internal referral	External referral	LP for *BRAF* & *KRAS*
Number of patients				
Total (*n*)	168	81	87	117
Male (*n*/%)	109/64.9	52/64.2%	57/65.5	76/65.0
Female (*n*/%)	59/35.1	29/35.8%	30/34.5	40/34.2
Age (mean/min/max)	61.7/33.2/92.4	59.8/33.2/82.9	63.5/38.0/92.4	61.6/36.0/92.4
Stage				
IV (*n*/%)	92/54.8	48/59.3	44/50.6	59/50.4
III (*n*/%)	25/14.9	19/23.5	6/6.9	18/15.4
II (*n*/%)	8/4.5	7/8.6	1/1.2	4/3.4
I (*n*/%)	5/2.9	5/6.2	0/0.0	5/4.3
n.a. (*n*/%)	38/22.6	2/2.5	36/41.4	31/26.5
Number of patients with *n* LP				
1 (*n*/%)	131/77.9	64/79.0	67/77.0	88/75.2
2 (*n*/%)	22/13.1	8/9.9	14/16.1	15/12.0
3 (*n*/%)	6/3.6	4.9	2/2.3	6/5.1
> 3 (*n*/%)	9/5.4	5/6.2	4/4.6	9/7.7
Number of LP requested				
Total (*n*)	243	115	128	155
2016 (*n*/%)	15/6.2	7/6.1	8/6.3	5/3.2
2017 (*n*/%)	60/24.7	14/12.2	46/35.9	31/20.0
2018 (*n*/%)	68/28.0	34/29.6	34/26.6	63/40.6
2019 (*n*/%)	51/21.0	33/28.7	18/14.1	39/25.2
2020 (*n*/%)	43/17.7	23/20	20/15.6	16/10.3
2021 (*n*/%)	6/2.47	4/3.5	2/1.6	1/0.6
Treatment at time of LP				
Naive (*n*/%)	21/8.6	18/15.7	3/2.3	18/11.6
No treatment (*n*/%)	23/9.5	18/15.7	5/3.9	14/9.0
Chemotherapy (*n*/%)	48/19.8	31/27.0	17/13.3	32/20.6
Anti‐VEGF (*n*/%)	39/16.1	21/18.3	18/14.1	21/13.5
Anti‐EGFR (*n*/%)	38/15.6	4/3.5	34/26.6	19/12.3
Immune checkpoint (*n*/%)	5/2.1	4/3.5	1/0.8	5/3.2
BRAFi/MEKi/anti‐EGFR (*n*/%)	3/1.2	3/2.6	0/0.0	3/1.9
Radiation (*n*/%)	1/0.4	1/0.9	0/0.0	1/0.6
n.a. (*n*/%)	65/26.8	15/13.0	50/39.1	42/27.1
Indication for LP				
MRD (*n*/%)	22/9.1	20/17.4	2/1.6	17/11.0
Treatment selection (*n*/%)	107/44.0	70/60.9	37/28.9	73/47.1
Monitoring (*n*/%)	39/16.1	6/5.2	33/25.3	20/12.9
n.a. (*n*/%)	75/30.9	19/16.5	56/43.8	45/29.0
Clinical decision based on LP				
Total (yes/no/n.a.)	150/17/76	82/14/19	68/3/57	96/14/45
2016 (yes/no/n.a.)	6/4/5	2/4/1	4/0/4	2/3/0
2017 (yes/no/n.a.)	29/6/25	8/6/0	21/0/25	12/4/15
2018 (yes/no/n.a.)	40/4/24	26/1/7	14/3/17	39/4/20
2019 (yes/no/n.a.)	42/2/7	27/2/4	15/0/3	31/2/6
2020 (yes/no/n.a.)	29/1/12	16/1/6	13/0/6	12/1/3
2021 (yes/no/n.a.)	4/0/2	3/0/1	1/0/1	0/0/1

### 3.2 Liquid profiling requests and use for clinical decision making

In 2016, blood‐based analysis of *RAS* and *BRAF* mutations in the circulation of CRC patients was introduced as routine clinical diagnostics at UMM. The test was offered free of charge to treating physicians and patients, as there was no possibility of reimbursement by health insurance companies at that time. Notably, the number of tests ordered in 2016 remained quite low, with a total of 15 LPs requested. Comparing 2017 to 2020, the number of LPs ordered first increased slightly, then stagnated and finally decreased by more than 30% from 2018 to 2020. However, comparison of internal and external referral shows that the number of LPs requested for UMM patients increased over all years excepting 2020, whereas tests requested from external hospitals decreased from 2017 onward (Table 1).

In cases where the indication of LP was reported, the majority of tests were ordered for therapy selection (44.0%), followed by monitoring of targeted therapy (16.1%) and MRD diagnostics (9.1%). Comparing 2017 to 2020, the number of LPs requested for therapy monitoring increased from 1.6% to 12.5%, for MRD diagnostics from 5.7% to 28.1%, and for treatment selection from 47.1% to 59.4%, respectively. Interestingly, substantial differences between internal and external referrals can be noted. Although treatment selection accounts for 60.9% of LPs requested for UMM patients, it represents LP indication in only 28.9% of external requests. A total of 74 LP requested for treatment selection were considered for clinical decision making. In 58/74 cases, tissue biopsy results were unavailable, so treatment selection was guided solely by LP. Specifically, *RAS* wild‐type status led to initiation of anti‐EGFR antibody treatment in 56.9% of cases; such treatment was stopped or never initiated in 36.2% of cases due to an identified *KRAS* mutation; and BRAFi therapy was initiated in 6.9% of patients due to a *BRAF* mutation identified by LP.

In addition, the clinical utility of ctDNA as part of routine management of CRC patients could be evaluated in 168/243 cases. Comparing 2017 to 2020, the number of LP results considered for clinical decision making increased from 48% in 2017 to 69% in 2020 or from 82.9% to 96.7% in cases with available information. For external requests, almost all tests were considered by treating physicians for clinical decisions. For internal referrals, over 50% of LPs were not considered for patient therapy adjustments in 2016. This number decreased to 4.3% in 2020.

Detailed information about tests ordered per year and the respective clinical indication is provided in Table 1 and Table S1.

### 3.3 Assessment of *RAS* and *BRAF* mutational status

In recent years, a total of 237 LP tests for CRC patients were ordered for *RAS* and 162 for *BRAF*, while in 155 cases *RAS* and *BRAF* analyses were requested simultaneously.

*KRAS*, *NRAS*, and *BRAF* mutations were detected in 25.9%, 0.7%, and 17.6% of tissue samples and in 29.1%, 6.8%, and 9.9% of evaluable plasma samples, respectively. A summary of the mutational distribution is provided in Table 2. *KRAS* codon 12 sequence variations were identified in 53/73 (72.6%) *RAS*‐mutated LP samples, followed by *NRAS* codon 61 mutations at 16.4% and *KRAS* codon 13 and 61 alterations at 10.9% each. Overall, no genetic alterations in *KRAS* or *NRAS* codon 59 or *NRAS* codon 146 could be identified. Regarding the frequency of *KRAS* and *BRAF* mutations, no significant differences between tissue and blood‐based testing could be revealed (Fisher's exact, *KRAS P* = 0.57; *BRAF P* = 0.13). However, *NRAS* variations occurred significantly more frequently in LP samples than in tissue samples (0.7% vs 6.8%, Fisher's exact *P* = 0.004). In this context, it is worth noting that 90.5% (38/42) of *KRAS*, 100% (1/1) of *NRAS*, and 77.8% (14/18) of *BRAF* variations occurred mutually exclusively in tissue, and that concomitant *BRAF*/*KRAS* or *BRAF*/*NRAS* mutations were identified in 9.5% (4/42) of mutation‐positive tissue samples, whereas no concomitant *KRAS*/*NRAS* alterations were identified. Compared to tissue, concomitant *KRAS*/*NRAS* mutations were observed in 10/69 (14.5%) *RAS*‐mutated LP samples. Unfortunately, clinical information regarding type of therapy at time of LP was only available in 4 cases, and these patients had all already received multiple therapies. LP confirmed concomitant *BRAF*/*RAS* tissue mutation in all four cases, while identifying an additional *NRAS* mutation in 2/4 cases.

**Table 2:** Results of LP.

	Total	Internal referral	External referral	LP for *BRAF* & *KRAS*
	(*n* = 243 LP)	(*n* = 115 LP)	(*n* = 128 LP)	(*n* = 155)
Tissue‐based testing				
Tissue results available (*n*/%)	162/243 (66.7%)	93/115 (80.9%)	69/128 (53.9%)	103/155 (66.5%)
*KRAS* mutant	42/162 (25.9%)	36/93 (38.7)	6/69 (8.7%)	28/103 (27.2%)
*KRAS* codon 12/13	29/156 (18.6%)	28/92 (30.4%)	1/64 (1.6%)	19/101 (18.8%)
*KRAS* codon 59/61	1/155 (0.6%)	1/91 (1.1%)	0/64 (0.0%)	1/101 (1.0%)
*KRAS* codon 146	6/155 (3.9%)	6/91 (6.6%)	0/64 (0.0%)	6/101 (5.9%)
*NRAS* mutant	1/142 (0.7%)	0/79 (0.0%)	1/63 (1.6%)	0/91 (0.0%)
*BRAF* V600 mutant	18/102 (17.6%)	15/76 (19.7%)	3/26 (11.5%)	14/70 (20.0%)
MSI	6/103 (5.8%)	6/74 (8.1%)	0/29 (0.0%)	5/64 (7.8%)
*KRAS* mutually exclusive	38/162 (23.5%)	33/93 (35.5%)	5/69 (7.2%)	25/103 (24.3%)
*NRAS* mutually exclusive	1/142 (0.7%)	0/79 (0.0%)	1/63 (1.6%)	0/91 (0.0%)
*KRAS* and *NRAS* concomitant	0/142 (0.0%)	0/79 (0.0%)	0/63 (0.0%)	0/91 (0.0%)
*BRAF* mutually exclusive	14/102 (13.7%)	12/76 (15.8%)	2/26 (7.7%)	11/70 (15.7%)
*BRAF* and *RAS* concomitant	4/93 (4.3%)	3/69 (4.3%)	1/24 (4.2%)	3/62 (4.8%)
Plasma‐based testing				
Samples *RAS* determined	237/243 (97.5%)	113/115 (98.3%)	124/128 (96.9%)	155/155 (100%)
Samples *BRAF* determined	162/243 (66.7%)	86/115 (74.8%)	76/128 (59.4%)	155/155 (100%)
Matching to tissue	19/243 (7.8%)	19/115 (16.5%)	0/128 (0.0%)	16/155 (10.3%)
*KRAS* mutant	69/237 (29.1%)	43/113 (38.1%)	26/124 (21.0%)	44/155 (28.4%)
*KRAS* codon 12 mutant	53/237 (22.4%)	33/113 (29.2%)	20/124 (16.1%)	32/155 (20.6%)
*KRAS* codon 13 mutant	8/237 (3.4%)	5/113 (4.4%)	3/124 (2.4%)	5/155 (3.2%)
*KRAS* codon 59 mutant	0/237 (0.0%)	0/113 (0.0%)	0/124 (0.0%)	0/155 (0.0%)
*KRAS* codon 61 mutant	8/236 (3.4%)	4/112 (3.6%)	4/124 (3.2%)	6/154 (3.9%)
*KRAS* codon 117 mutant	1/237 (0.4%)	1/113 (0.9%)	0/124 (0.0%)	1/155 (0.6%)
*KRAS* codon 146 mutant	6/237 (2.5%)	4/113 (3.5%)	2/124 (1.6%)	6/155 (3.9%)
*NRAS* mutant	16/237 (6.8%)	6/113 (5.3%)	10/124 (8.1%)	9/155 (5.8%)
*NRAS* codon 12 mutant	4/236 (1.7%)	1/112 (0.9%)	3/124 (2.4%)	3/154 (1.9%)
*NRAS* codon 13 mutant	1/237 (0.4%)	1/113 (0.9%)	0/124 (0.0%)	1/155 (0.6%)
*NRAS* codon 59 mutant	0/237 (0.0%)	0/113 (0.0%)	0/124 (0.0%)	0/155 (0.0%)
*NRAS* codon 61 mutant	12/237 (5.1%)	6/107 (5.3%)	6/124 (4.8%)	6/155 (3.9%)
*NRAS* codon 117 mutant	1/237 (0.4%)	0/113 (0.0%)	1/124 (0.8%)	0/155 (0.0%)
*NRAS* codon 146 mutant	0/237 (0.0%)	0/113 (0.0%)	0/124 (0.0%)	0/155 (0.0%)
*BRAF* V600 mutant	16/162 (9.9)	12/86 (14.0%)	4/76 (5.3%)	16/155 (10.3%)
*KRAS* mutually exclusive	55/237 (23.2%)	36/113 (31.9%)	19/124 (15.3%)	35/155 (22.6%)
*NRAS* mutually exclusive	4/237 (1.7%)	0/113 (0.0%)	4/124 (3.2%)	2/155 (1.3%)
*KRAS* and *NRAS* concomitant	10/237 (4.2%)	4/113 (3.5%)	6/124 (4.8%)	5/155 (3.2%)
*BRAF* mutually exclusive	12/162 (7.4%)	9/86 (10.5%)	3/76 (3.9%)	12/155 (7.7%)
*BRAF* and *RAS* concomitant	4/162 (2.5%)	3/86 (3.5%)	1/76 (1.3%)	4/155 (2.6%)
MAF				
*KRAS* MAF (min/max/median/IQR)^a^	0.025/24.0/0.62/3.47	0.03/24.0/0.5/3.22	0.025/20.0/0.95/5.83	0.025/20.0/0.6/3.15
*NRAS* MAF (min/max/median/IQR)^a^	0.02/9.4/0.15/0.24	0.05/1.0/0.19/0.26	0.02/9.4/0.13/0.20	0.05/1.0/0.09/0.25
*BRAF* MAF (min/max/median/IQR)^a^	0.24/59.0/18.1/30.4	1.0/59.0/20.4/28.4	0.24/35.3/9.0/19.57	0.24/59.0/20.9/29.5
Concordance^b^				
Positive agreement	5/7 (71.4%)	5/7 (71.4%)	n.a.	5/6 (83.3%)
Negative agreement	39/41 (95.1%)	39/41 (95.1%)	n.a.	36/38 (94.7%)
Overall agreement	44/48 (91.7%)	44/48 (91.7%)	n.a.	41/44 (93.2%)
Kappa	0.666	0.666	n.a.	0.730
SE of kappa	0.155	0.155	n.a.	0.147
95% CI	0.361–0.970	0.361–0.970	n.a.	0.440–1.000
Emerging mutation under therapy^c^				
*KRAS*	14/162 (8.6%)	9/93 (9.7%)	5/69 (7.2%)	10/103 (9.7%)
*NRAS*	5/142 (3.5%)	1/79 (1.3%)	4/63 (6.3%)	4/91 (4.4%)
*BRAF*	0/102 (0.0%)	0/76 (0.0%)	0/26 (0.0%)	0/70 (0.0%)

^a^ MAF was calculated for mutant samples.

^b^ Determined for simultaneously obtained tissue and blood samples.

^c^ Determined if tissue results were available.

The majority of LPs were requested for stage IV cancer patients (146/243), with LP detecting a mutation in 33.6% (49/146). Overall, 23% (56/243) of LPs were requested for earlier CRC stages. For stage I‐III cancer patients, either a *RAS* or *BRAF* mutation was detected in 24/56 (42.9%) of requested LPs; for stage I–II patients, LP was positive in 9/22 (40.9%) of cases, and in 1/5 (20%) of cases for stage I.

In general, the mean interval between tissue‐ and plasma‐based genetic testing was 664 days (median: tissue biopsy 370 days before LP; minimum: tissue biopsy 2904 days before LP; maximum: tissue biopsy 665 days after LP). If the time interval between both test modalities was < 30 days, tissue biopsy and LP were considered timely matched. In total, a timely matched tissue biopsy was available for 19 (11.3%) patients, with tissue biopsy analyzed on average 4.94 days later (median: tissue biopsy 3 days after LP; minimum: tissue biopsy 8 days before LP; maximum: tissue biopsy 26 days after LP). Concordance analysis for these tests resulted in an overall agreement of 91.7%. 5/7 tissue mutations were detected in ctDNA, resulting in a sensitivity of 71.4%. 2/41 LP‐positive/tissue‐negative results were reported, leading to a specificity of 95.1%. Establishing concordance between both methods yielded a Cohen's kappa of 0.666, indicating substantial agreement between tissue testing and LP.

### 3.4 Evaluation of mutant allele frequency

**Fig. 2:**
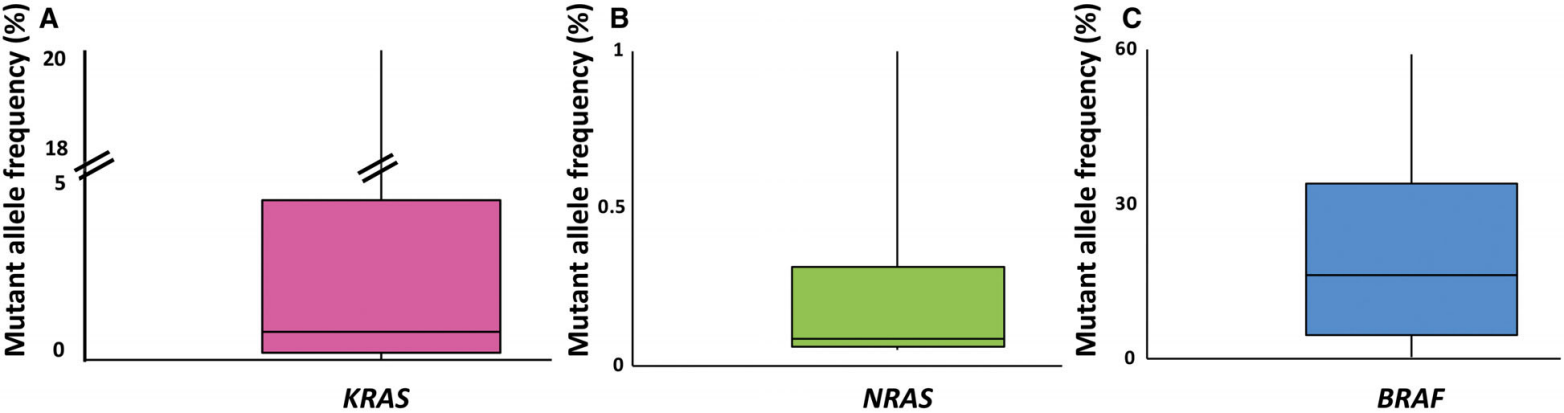
ctDNA fraction of LP. Box plots of the MAF (y‐axis) of ctDNA determined for (A) *KRAS* (*n* = 69 mutant patient samples), (B) *NRAS* (*n* = 16 mutant patient samples), and (C) *BRAF* (*n* = 16 mutant patient samples) are depicted. All patient samples were determined by single measurement without duplicates and are based on analysis of total cfDNA isolated from 3 mL of plasma. The lower and upper lines of each box correspond to the 25th and 75th percentile, respectively. The horizontal line inside each box indicates the median, the whiskers the extreme values measured.

ctDNA was detectable in 29.1%, 6.8%, and 9.9% of LPs for *KRAS*, *NRAS*, and *BRAF*, respectively. The estimated median ctDNA fractions as well as the interquartile ranges are depicted in Fig. 2. Interestingly, the lowest median MAF was observed for *NRAS* at 0.15%, followed by *KRAS* (0.62%) and *BRAF* (18.1%). Importantly, 53.2% of all measured ctDNA fractions were below 1%, 46.8% below 0.5%, 21.1% below 0.1%, and 8.3% below 0.05%. However, there were no significant differences between patients on active therapy and those who were off treatment or treatment‐naïve (*t*‐test, *KRAS P* = 0.54, *BRAF P* = 0.63). Comparing patients with a high tumor burden [progressive disease (PD) or treatment‐naïve] to those with a low tumor burden (SD, PR or CR), a significant difference in the ctDNA fraction emerged for *BRAF* (22.2% vs 4.7%, *P* < 0.05), while no significant differences were observed for *KRAS* (3.0% vs 2.3%, *P* = 0.63) and *NRAS* (0.09% vs 0.23%, *P* = 0.10).

### 3.5 Evaluation of liquid profiling for follow‐up of CRC patients

**Fig. 3:**
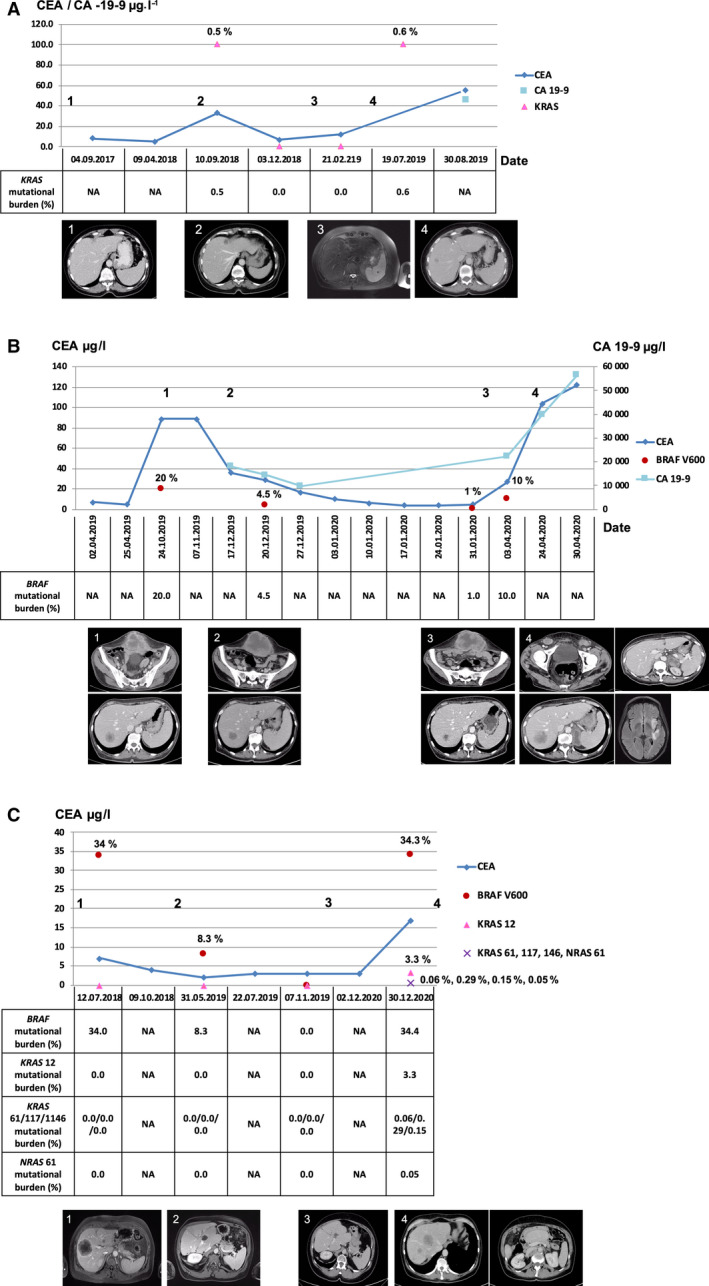
ctDNA monitoring during clinical disease course. Serial monitoring of ctDNA for three exemplary CRC patients is provided. MAF determined at different time points is displayed as triangles (*KRAS*) or dots (*BRAF*), and is compared to the level of the protein tumor markers CEA (dark blue line, µg·L^−1^) and CA 19‐9 (light blue square, µg·L^−1^). Additionally, the ctDNA mutational burden is provided in tabular view below each diagram. Exemplary imaging findings (CT or MRI) of respective tumor lesions are shown, and the time points of imaging assessment are indicated as numbers in the diagram. (A) 64‐year‐old women with RAS/BRAF wild‐type CRC of the cecum diagnosed in 08/2017 (1). After hemicolectomy followed by adjuvant chemotherapy, the patient had hepatic progression accompanied by an emerging *KRAS* mutation in 09/2018 (2). After partial liver resection, LP turned negative and CT and a dedicated MRI of the liver revealed CR (3). In 07/2019, hepatic metastasis recurred and the *KRAS* codon 13 mutation reappeared on LP testing (4). (B) 73‐year‐old women with *BRAF* V600E positive adenocarcinoma of the colon. After hemicolectomy (04/2019) and adjuvant chemotherapy (Oxaliplatin/Capecitabine), CT scan in 10/2019 revealed an abdominal wall lesion and a hepatic metastasis and LP showed a *BRAF* mutation (1). After four cycles of FOLFIRI, the patient progressed in 01/2020 (2) and was therefore placed on Encorafenib/Binimetinib/Cetuximab combination therapy. During follow‐up, MAF in LP decreased to 1%, as did the abdominal and hepatic lesions in CT (3). Despite surgical treatment of the abdominal mass, *BRAF* ctDNA level increased to 10%. Shortly thereafter, CT/MRI scans showed multiple newly hepatic, cerebral and lymph node metastases (4). (C) 49‐year‐old woman with hepatic metastatic rectum carcinoma. A *BRAF* mutation was identified in tissue and LP (1). After therapy initiation, imaging and LP indicated a PR (2) and CR (3). In 12/2020, the patient progressed with multiple variations identified in LP. PD was later confirmed by imaging (4).

A total of 111 LPs were ordered during follow‐up of CRC patients for monitoring of targeted therapy, MRD assessment, or treatment selection in case of disease progression. On average, 3.0 samples were analyzed per patient, with a median follow‐up of 336 days (25–75%: 105–427 days). The presence of a *RAS* or *BRAF* mutation was confirmed in 35 cases, and therapy selection was directly initiated accordingly in 11/15 cases. Of note, in 19 cases, sequence variation not previously detected in tumor tissue that emerged under therapy (five *NRAS*, 14 *KRAS*). In 13 cases, disease progression was detected by imaging during follow‐up. In eight/13 patients, increasing ctDNA levels indicating PD were identified. In six cases, ctDNA level rebounded at time of imaging, and in two cases, an increase in ctDNA level was detected prior to imaging. In four cases, however, LP detected no PD in *RAS* and *BRAF* wild‐type tumor; in one case, LP detected no PD, although the primary tumor was *RAS* mutated. Here, progression of peritoneal affection was diagnosed by imaging. To provide insight into the clinical utility of LP for CRC patient management in daily clinical routine and as a complementary diagnostic tool, three exemplary cases are described below (Fig. 3).

The first case (Fig. 3A) is a 64‐year‐old woman with *RAS* and *BRAF* wild‐type adenocarcinoma of the cecum, first diagnosed in 08/2017. After hemicolectomy, she was started on adjuvant chemotherapy (Oxaliplatin/Capecitabine) for 3 months. Follow‐up (09/2018) revealed a *KRAS* codon 13 mutation (0.5%), which corresponded with CT findings of two liver metastases. Based on finding of PD by LP and imaging, a partial liver resection was performed. The patient achieved CR and LP turned negative, indicating the success of treatment. However, disease recurrence was detected by imaging and LP (*KRAS* codon 13 0.6%) in 01/2019. As PD was identified by LP and imaging, with the *KRAS* positivity preventing anti‐EGFR antibody treatment, the patient was started on FOLFIRI and Bevacizumab.

The second case (Fig. 3B) represents a 73‐year‐old woman with *BRAF* V600E‐positive adenocarcinoma of the colon. After hemicolectomy (04/2019) and 5 months of adjuvant chemotherapy (Oxaliplatin/Capecitabine), a CT scan in 10/2019 revealed an abdominal wall lesion and a hepatic metastasis. The finding of PD was confirmed by *BRAF* positivity (20%) in LP. The patient progressed after 4 cycles of FOLFIRI and was therefore placed on Encorafenib/Binimetinib/Cetuximab combination therapy based on the results revealed by LP (*BRAF* positivity, *RAS* negativity). During follow‐up, MAF in LP decreased to 1%, as did the abdominal and hepatic lesions in CT (01/2020). Despite surgical removal of the abdominal mass, *BRAF* ctDNA level increased to 10%. Shortly thereafter, CT/magnetic resonance imaging (MRI) scans showed multiple metastases (04/2020). Although LP indicated PD, the unchanged mutational profile did not allow for treatment adjustment. Here, extensive LP covering a gene panel may have been of additional benefit.

Figure 3C shows the case of a 49‐year‐old woman with *BRAF* V600E‐mutated hepatic metastasized rectal cancer. *BRAF* positivity (34%) was confirmed by LP before initiation of therapy (FOLFIRI followed by FOLFIRI/Bevacizumab as well as metastasectomy) in 07/2018. Imaging revealed CR, and LP became negative (11/2019). After 7 months, progression occurred and the patient was finally placed on encorafenib/cetuximab, according to the results of LP. LP was performed in 12/2020 and showed *BRAF* positivity (34.3%) along with multiple newly emerged *RAS* mutations. The newly emerged *RAS* mutations detected by LP were used for the clinical decision to stop Cetuximab and start the patient on FOLFIRI and Aflibercept.

## 4 Discussion

The clinical utility of LP is versatile and has been proven in translational studies, publications on the implementation and diagnostic performance of LP in clinical practice remain limited, leaving its value beyond clinical trials elusive [30, 31]. To address this gap, the translation of LP into standard health care, surveillance, and clinical decision making for CRC patients were assessed through a retrospective analysis of real‐world, single‐center data obtained in the context of clinical care since 2016.

Here, a steady increase of LP requests for UMM patients was noted, except in 2020. This decline is most likely due to the ongoing corona virus disease 2019 pandemic and the associated interim closure of hospital outpatient departments, as well as patients' reluctance to consult physicians as a result of the potentially higher risk of infection in the hospital setting. In contrast, external inquiries showed a continuous decrease since 2017. This could be explained by the dissemination of LP testing within Germany, and thus LP testing in one's own hospital laboratory. Overall, there was a very restrained inquiry behavior on the part of clinicians, especially initially. The lack of reimbursement is often referred as a key limitation on translating LP into daily care [25]. However, all tests performed as part of standard care within this retrospective evaluation of real‐world clinical data were offered free of charge to clinicians and patients—reimbursement was available as part of outpatient specialized care, under a special agreement with certain health insurers, or through UMM's full coverage of costs. Thus, reimbursement had no impact on request behavior. As such, it is more likely that clinicians were not initially fully convinced of the clinical utility of LP. This could be due to the lack of large‐scale prospective clinical trials, the lack of quality and performance standards for ctDNA analysis, the lack of integration of LP into current clinical workflows, or the still insufficient clinical validity and utility for most ctDNA assays according to college of American Pathologists [32]. This skepticism of clinicians is also reflected in the request indication for LP. Initially, the majority of LP was requested concurrently with tissue‐based testing and was not used for clinical decision making. Over the years, LP has been integrated into clinical workflows and used to follow‐up patients or to support therapeutic decisions—underscoring its gradual acceptance by clinicians.

Analysis of these real‐world data revealed a frequency of oncogenic driver mutations in *RAS* and *BRAF* in 26% and 10% of LP samples, consistent with other studies [33, 34]. In cases where temporally matched tissue biopsy results were available, there was a high overall agreement of 91.7%. No significant difference existed between tissue‐ and plasma‐based testing, except for *NRAS*. This high level of concordance is comparable to that reported in translational studies [19, 35] and shows that high‐quality, reliable analyses can be performed even under routine conditions in standard care. Compared to the specificity of 95%, the sensitivity of 71% was moderate, indicating that sufficient amounts of ctDNA are not shed at all times. These false‐negative results might be mitigated through serial monitoring or biopsy confirmation, if available [36]. Notably, in contrast to tissue, concurrent *KRAS* and *NRAS* mutations were observed in 14.5% of *RAS*‐mutated LP samples, which could be explained by bypass mechanisms of acquired resistance of certain subclones to targeted therapy [37]. *BRAF* and *RAS* mutations rarely occur concomitantly and are therefore considered mutually exclusive [2, 34]. Nevertheless, concurrent findings in LP have been reported previously [38]. This highlights that by tissue biopsy alone the number of cases with multiple driver mutations is underestimated and needs to be reassessed by LP during the course of treatment to provide appropriate guidance for therapeutic decisions.

Interestingly, 23% of all LPs were requested for stage I–III CRC patients, although LP is recommended in Germany exclusively for stage IV CRC patients for treatment selection. Surprisingly, over 40% of these LPs were found to have either a *BRAF* or *RAS* mutation. This high frequency of LP positivity can likely be explained by the fact that most of these patients were treatment naïve—ctDNA levels might accordingly be higher than in stage IV CRC patients under therapy. The use of LP in earlier stages of cancer is currently being investigated in prospective clinical trials such as CIRCULATE (AIO‐KRK‐0217) or COBRA (NRG‐GI005) [10, 39, 40, 41], for example, to assess the need for adjuvant chemotherapy. Based on the results of these standard‐of‐care LP tests, LP may be suitable to determine the mutational status also in earlier stages, although in these cases cfDNA levels should be assessed in order to minimize the risk of false‐negative test results. Although the use of ctDNA for screening has also been addressed recently [42, 43], the lack of studies identifying diagnostic procedures for specific subgroups of patients at increased risk has so far hindered the use of LP for this type of diagnosis. Future results of prospective, integrative studies will indicate whether LP can be used for this purpose in CRC patients as well.

The majority of LPs were requested for stage IV cancer patients (146/243), with 33.6% (49/146) having a mutation detected by LP. Overall, 23% (56/243) of LPs were requested for earlier CRC stages. For stage I‐III cancer patients, either a *RAS* or *BRAF* mutation was detected in 24/56 (42.9%) of requested LPs, while LP was positive in 9/22 (40.9%) cases for stage I–II patients, and for 1/5 (20%) stage I cases.

As the mean MAF in LP samples analyzed as part of standard care was < 0.5% in almost 50% of cases and < 0.1% in over 20%, the urgent need for mandatory, accurate, sensitive, and reliable ctDNA diagnostic and performance standards becomes obvious. In particular, compared to the high MAF of *BRAF*‐mutated tumors, which are known to be more aggressive, the median MAF of *NRAS* mutations was extremely low. This could be because *NRAS* mutations frequently occurred concomitantly to *KRAS* variations in patients under treatment, potentially indicating the emergence of resistant subclones and thus one of the main indications for performing LP. This is further supported by the significantly higher frequency of *NRAS* mutations in LP compared to tissue biopsy. If the occurrence of *NRAS* mutations represents a resistance mechanism, the need to include LP for monitoring response to targeted therapies on a regular routine basis becomes obvious. Apart from the need for sensitive assays, another issue resulting from emerging resistance mutations with extreme low MAFs is their clinical relevance. To date, there is no consensus regarding clinically relevant thresholds that warrant treatment breaks or re‐challenge [36]. Indicative of the clinical relevance of even these highly underrepresented mutations is the report by Parseghian et al. [44] that patients with persistent *RAS*‐mutated clones do not benefit from Cetuximab re‐challenges regardless of their MAF, and the study by Aggarwal et al. [45] showing a significant clinical response in non‐small‐cell lung cancer patients for low MAF resistance mutations. Nonetheless, the lack of clinically relevant cut‐offs renders clinicians uncertain of the optimal use of LP for clinical decision making, and is an issue further studies should address to enable a successful translation of LP into standard care for CRC patients in the long term.

The development of targeted therapies fundamentally changed the management of CRC patient and improved outcomes. However, the efficacy of targeted therapy is compromised by emerging resistance mutations. As response assessment relies primarily on imaging, which does not reflect clonal tumor evolution at the molecular level, LP is an attractive complementary diagnostic tool for monitoring targeted therapy and MRD [8, 9, 11, 36, 46]. This is further supported by the clinical data presented here and the three exemplary case reports. In general, ctDNA levels mirrored radiographic findings, with a decline in ctDNA MAF indicating response to therapy and an increase indicating recurrence. One case excepted, disease progression of *RAS*‐mutated tumors was detected by LP prior to or concurrently with imaging. In this case, the metastatic spread to the peritoneum was missed by ctDNA analysis as described previously [36, 47]. Although ctDNA is reported to have a shorter lead‐time of up to 10 months to PD detection compared to imaging [48], LP preceded imaging in only two cases in this real‐world setting. The most likely explanation for these discrepant findings is the choice of timing for LP testing. Radiographic and molecular responses have different kinetics; therefore, the intervals for assessment should be appropriately defined for each diagnostic type. This has not been the case in clinical practice. Rather, LP has been requested in addition to protein tumor markers or imaging‐based follow‐up, although not always and not regularly, resulting in a severe limitation of the clinical utility of LP. Hence, there is an urgent need to define optimal time points and incorporate these into guidelines, as others have called for [36, 46].

Although this retrospective analysis of real‐world data provides first insights into the translation of LP into CRC patients standard care, it has several limitations, such as the limited number of LP tests requested in recent years, the *post hoc* exploratory analysis, and the evaluation of clinical information based on medical reports provided in the hospital information system and/or via physician notes. Overall, the conclusions drawn from this retrospective evaluation are compromised by the limited number of LPs, the missing clinical information for a substantial number of patients, the limited number of LPs ordered for early‐stage CRC patients, and the heterogeneity of clinical indications. However, because results of a retrospective evaluation of clinical data obtained in routine clinical practice, rather than a retrospective study, are reported, these limitations reflect the reality and challenges of LP testing as part of standard care.

## 5 Conclusion

These results highlight the value of LP for CRC patient management in daily clinical practice. They demonstrate that LP is already being used for clinical decision making, that the results may alter therapeutic decisions, and that they could impact clinical outcomes. However, the establishment of quality standards and short turnaround times, as well as regular exchange with clinicians, are prerequisites for successful clinical translation. In addition, the definition of clinically relevant cutoffs and optimal time points for ctDNA testing is urgently needed. LP should be considered a complimentary diagnostic tool, with further studies warranted to elucidate the clinical utility of an integrative diagnostic approach for clinical decision making in CRC patient management.

## Conflict of interest

The authors declare no conflict of interest.

## Author contributions

VH and MN designed the study. AD and LM performed assays. MH, VH, RDH, UMM, RPR, and MFF were responsible for data collection. MFF evaluated imaging findings. MH, VH, and MN were responsible for evaluation of LP results. VH and VA were responsible for biostatistics analyses. VH and MH were responsible for interpretation of data. MH and VH prepared tables and figures. VH and MH drafted the manuscript. All authors contributed to revision of the manuscript and approved it for submission.

### Peer Review

The peer review history for this article is available at https://publons.com/publon/10.1002/1878‐0261.13156.

## Supporting information


**Table S1**. Overview of clinical and analytical data of all patients.

## Data Availability

The data that support the findings of this study are available in the supplementary material of this article; any further data that support the findings of this study are available from the corresponding author (verena.haselmann@umm.de) upon reasonable request.
